# New-Onset Myasthenia Gravis Associated with SARS-CoV-2 Infection: A Systematic Review

**DOI:** 10.3390/life16071100

**Published:** 2026-06-30

**Authors:** Amalia L. Călinoiu, Alexandra Mincă, Claudiu C. Popescu, Ileana M. Vodă, Alexandra M. Cristea, Dragoș I. Mincă

**Affiliations:** 1 “Prof. Dr. Agrippa Ionescu” Emergency Clinical Hospital, 011356 Bucharest, Romania; 2 Public Health and Management Department, “Carol Davila” University of Medicine and Pharmacy, 020021 Bucharest, Romania; 3 Rheumatology Department, “Carol Davila” University of Medicine and Pharmacy, 020021 Bucharest, Romania; 4 Bucharest University Emergency Hospital, 050098 Bucharest, Romania; 5 Pneumology I Department, Carol Davila University of Medicine and Pharmacy, 020021 Bucharest, Romania; 6 Marius Nasta Institute of Pneumology, 050159 Bucharest, Romania

**Keywords:** myasthenia gravis, COVID-19, SARS-CoV-2 infection, Long-COVID

## Abstract

This systematic literature review synthesizes available data from published case reports and case studies describing de novo myasthenia gravis (MG) following confirmed SARS-CoV-2 infection, focusing on clinical presentation, immunological characteristics, temporal relationship, management, and outcomes. The study addresses the following research question: What are the clinical, immunological, temporal, and outcome characteristics of de novo MG following SARS-CoV-2 infection based on published case reports and case studies? Following the PRISMA 2020 guidelines, the analysis included 44 studies describing 48 patients. The findings suggest that MG temporally associated with COVID-19 typically occurred within a relatively short latency period, with a median interval of 21 days. Most patients presented with generalized onset and were predominantly positive for AChR-Abs, consistent with the established clinical and serological profile of classical MG, in which AChR-Abs represent the most common subtype, particularly in generalized disease. MuSK and LRP4-positive cases were less frequent; ICU admission, myasthenic crisis, and respiratory failure were frequently reported. Importantly, most patients responded well to conventional MG therapies.

## 1. Introduction

Since 2020, when the World Health Organization (WHO) declared a pandemic, the outbreak of severe acute respiratory syndrome caused by Coronavirus 2 (SARS-CoV-2), an estimated 778 million confirmed infections and approximately 7.1 million related deaths have been reported worldwide to date [1]. Over the subsequent years, due to its high transmissibility, the novel disease(COVID-19) has rapidly disseminated across the globe [2,3]. Although the coronavirus disease 2019 (COVID-19) pandemic has formally ended, its sequelae persist, and studies continue to reveal novel associations between SARS-CoV-2 infection and different pathologies [4].

COVID-19 initially garnered attention for its predominant respiratory involvement [5], but evidence demonstrated that the virus can infect multiple organ systems [6]. SARS-CoV-2 infection has been linked to a wide spectrum of neurological complications, such as neuropathies, myopathies, MG and Guillain–Barré syndrome [7,8]. Notably, both the central and peripheral nervous systems seem to be involved [9,10,11]. The emergence of SARS-CoV-2 has raised concerns regarding its potential role in triggering autoimmune neuromuscular disorders [12]. As the medical community transitioned into the post-COVID-19 pandemic era, the focus has shifted toward understanding the long-term autoimmune consequences triggered by the virus, including the potential emergence of rare immune-mediated neuromuscular diseases [4].

Myasthenia gravis is an autoimmune neuromuscular disorder marked by fluctuating skeletal muscle weakness involving various muscle groups caused by the pathogenic action of specific autoantibodies, resulting in a reduction in functional acetylcholine receptors (AChR) and structural disruption of the neuromuscular junction [13]. The onset of MG associated with particular infectious events has been documented across multiple case series (CS), but the etiology remains unknown [14,15]. Furthermore, current data do not demonstrate a definitive association between MG and any specific infectious antecedent processes [15]. A possible association between COVID-19 and new-onset MG has been proposed, as well as the occurrence of myasthenic crisis, respiratory failure, and higher mortality, potentially mediated by an exaggerated inflammatory response [16]. Although cases were classified as de novo MG based on the absence of a previously documented diagnosis, the possibility that SARS-CoV-2 infection unmasked pre-existing subclinical disease cannot be excluded. Several biological mechanisms have been proposed to explain the temporal association between SARS-CoV-2 infection and MG onset. Viral infections may promote autoimmunity through mechanisms such as molecular mimicry, epitope spreading, bystander activation, and dysregulated B- and T-cell responses [14]. Since the emergence of SARS-CoV-2, reports have described both worsening of previously stable, established MG and the occurrence of newly diagnosed cases [17]. SARS-CoV-2 infection is characterized by marked immune activation, which may contribute to loss of self-tolerance and autoantibody production in susceptible individuals [18,19]. However, these mechanisms remain hypothetical and have not been definitively demonstrated in MG following COVID-19 [20].

Despite the increasing number of published reports describing MG occurring after SARS-CoV-2 infection, these are limited to individual case reports (CRs) and small case series (CS). Consequently, the clinical presentation, immunological profile, temporal relationship, and therapeutic outcomes of de novo MG in this context have not been systematically characterized. Moreover, distinguishing a true post-infectious autoimmune phenomenon from a concurrent temporal association remains challenging, particularly given the high global prevalence of SARS-CoV-2 infection. The heterogeneity of reported cases, variability in diagnostic confirmation, and inconsistent reporting of key clinical variables further limit the interpretability of the current literature. In this context, a systematic synthesis of individual patient-level data is needed to better delineate the clinical and biological features of MG developing after SARS-CoV-2 infection and to identify patterns that may support or refute a potential association.

Therefore, this systematic literature review (SLR) aims to synthesize available evidence from published CR and CS describing de novo MG following confirmed SARS-CoV-2 infection, focusing on clinical presentation, immunological characteristics, temporal relationship, management, and outcomes. The study addresses the following research question: *What are the clinical, immunological, temporal, and outcome characteristics of de novo MG following SARS-CoV-2 infection based on published CRs and CS?*

## 2. Materials and Methods

This review was conducted in accordance with the PRISMA 2020 guidelines (Preferred Reporting Items for Systematic Reviews and Meta-Analyses) [21]. The review protocol was prospectively registered in the PROSPERO database (CRD420261342180). The analysis included published CRs and CS describing SARS-CoV-2 infection followed by new-onset MG, while review articles and consensus statements were used for narrative review. Given the available data, predominantly derived from CRs and CS, this SLR was designed as a descriptive hypothesis-generating synthesis rather than establishing causality, and particular attention was paid to the temporal relationship between SARS-CoV-2 infection and MG onset.

### 2.1. Inclusion and Exclusion Criteria

In the SLR, six eligibility criteria were applied, all were required for a study to be included: studies reporting adult patients (≥18 years old); studies reporting new-onset MG defined as the first documented clinical diagnosis of MG occurring after SARS-CoV-2 infection, with no prior history or diagnosis of MG reported; studies in which COVID-19 infection was confirmed by polymerase chain reaction (PCR), rapid antigen testing, or serological assays; studies with a CR or CS design; studies published from December 2019 to October 2025; and studies published in English.

Also, the following four exclusion criteria were used: studies reporting cases of exacerbation of pre-existing MG temporally associated with COVID-19; editorials, commentaries and review articles without original case data (used only for narrative review); reports with insufficient information to confirm the absence of pre-existing MG or the diagnosis of both SARS-CoV-2 infection and MG; and epidemiological studies without individual patient-level data confirming temporal association between SARS-CoV-2 infection and MG onset.

### 2.2. Databases and Citations Search

An electronic search of five bibliographic databases, including PubMed^®^, Embase^®^, Ovid^®,^ Scopus^®^ and Web of Science^®^, was performed on 3 October 2025, for English-language publications using the keywords “myasthenia gravis” combined with “COVID-19”, “SARS-CoV-2”. The used strategy is reported in Table 1. Additionally, the reference lists of the included articles were reviewed for further eligible records.

To ensure comprehensive retrieval of relevant evidence, two complementary search strategies were developed: a broad strategy and a focused strategy, as follows. The broad search strategy aimed to maximize sensitivity by identifying all potentially relevant records in which MG and COVID-19 were jointly mentioned. The objective of the broad strategy was to capture the full spectrum of reports describing MG temporally associated with SARS-CoV-2 infection, thereby minimizing the risk of missing eligible cases. The focused search strategy was designed to increase specificity by additionally incorporating free-text terms referring to the first episode of MG (e.g., “new-onset,” “de novo,” “first presentation,” “initial presentation”). This strategy targeted studies explicitly describing MG developing for the first time after COVID-19. While narrower in scope, the focused strategy facilitated the identification of cases likely to represent true de novo MG temporally associated with SARS-CoV-2 infection. Both strategies were applied across the five aforementioned databases. The results were combined, exported, deduplicated, and screened according to predefined eligibility criteria.

### 2.3. Selection of Records

**Figure 1:**
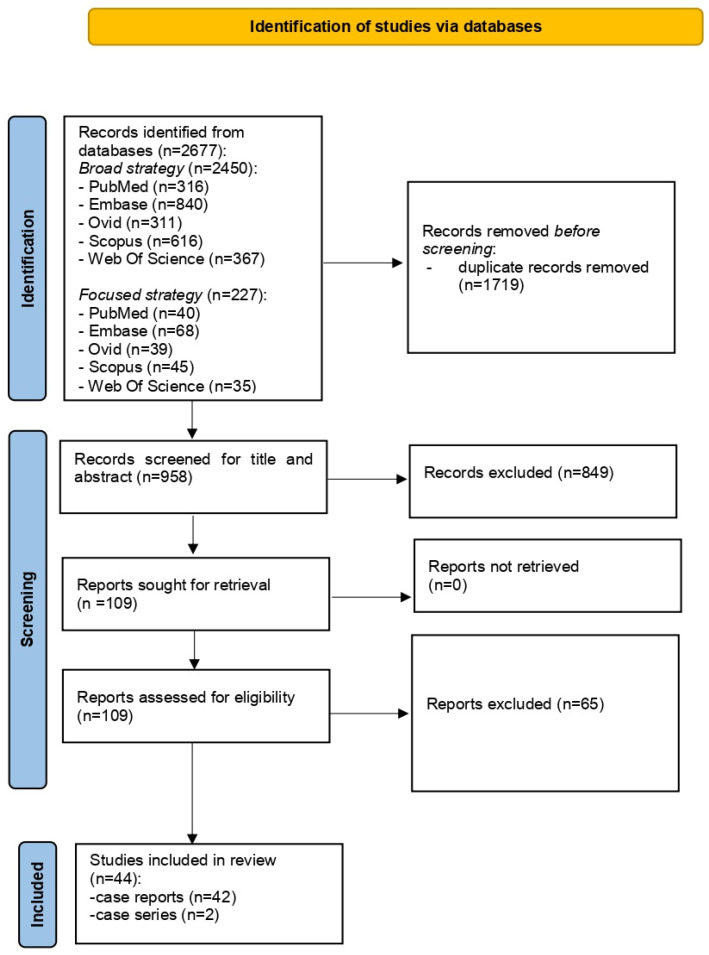
PRISMA 2020 flow diagram of the study selection process.

All records retrieved from the database searches and citation screening were imported into Mendeley Reference Manager for deduplication and subsequently transferred to Rayyan QCRI for screening. Titles, abstracts, and full texts were assessed independently by two reviewers according to the predefined eligibility criteria, with disagreements resolved through discussion or consultation with a third reviewer when necessary. Reasons for exclusion at the full-text stage were recorded in accordance with PRISMA guidelines. The study selection process was documented using a PRISMA 2020 flow diagram (Figure 1), and all included studies were grouped for synthesis according to study design.

### 2.4. Data Extraction

The selected studies were reviewed to extract the following variables for further analysis: study characteristics, patient demographics, medical history, COVID-19-related variables, MG clinical and diagnostic features, treatment, and outcomes. Data extraction and methodological quality assessment were performed independently by two reviewers, with disagreements resolved through discussion and, when necessary, consultation with a third reviewer. The Joanna Briggs Institute (JBI) critical appraisal tools for case reports and case series were used [22]. No automated tools were employed during data extraction or quality assessment.

## 3. Results

### 3.1. Study Selection and Characteristics

After removing duplicates, 958 unique records were obtained (Figure 1), and, following the screening of titles and abstracts, 109 articles were selected for full-text assessment. Full-text screening excluded 65 original articles [11,12,16,17,20,23,24,25,26,27,28,29,30,31,32,33,34,35,36,37,38,39,40,41,42,43,44,45,46,47,48,49,50,51,52,53,54,55,56,57,58,59,60,61,62,63,64,65,66,67,68,69,70,71,72,73,74,75,76,77,78] (Table 2); thus, 44 studies (CRs and CS), including 48 patients, were included in this SLR [79,80,81,82,83,84,85,86,87,88,89,90,91,92,93,94,95,96,97,98,99,100,101,102,103,104,105,106,107,108,109,110,111,112,113,114,115,116,117,118,119,120,121,122] (Table 3). Of the included studies, 42 were single-patient CRs (*n* = 42), while two were CS (*n* = 6) [114,123], highlighting the rarity of the occurrence and the limited level of evidence currently available. Regarding publication type, 15 studies were published as meeting abstracts (MA, 34.0%) [79,80,81,82,83,86,94,100,102,106,108,113,115,117,122] while 29 were full-length journal articles (JAs); all were published between 2020 and 2025 (Table 3). The studies originated from 18 countries; the United States was the most frequently represented (*n* = 16, 36.4%) [79,81,82,83,90,93,94,100,101,108,113,114,115,116,117,122], followed by Italy (*n* = 5, 11.4%) and India (*n* = 4, 9.1%). Sex data were available for 47 of the 48 patients (97.9%), with a slight female predominance (*n* = 24, 51.0%). Age was reported for all patients, with a median of 61 years (Table 3). The year of onset of MG symptoms following SARS-CoV-2 infection was reported in 16 studies (36.4%), with the earliest onset in 2020 and the latest in 2023. The most frequently reported year of symptom onset was 2020, accounting for 10 studies (62.5%, Table 3).

### 3.2. Baseline Characteristics and Comorbidities

Baseline clinical characteristics were variably reported across the included studies. The reported associated autoimmune diseases (ADs) were heterogeneous and did not reveal a consistent pattern. A personal history of ADs was identified in 13.2% (*n* = 5) of patients with available data (*n* = 38) [81,92,101,107,119], while a family history of ADs was infrequently documented (*n* = 2) [96,110]. Similarly, neurological comorbidities (*n* = 5, 15.2% from the available data) [81,90,95,101,116] and prior or active neoplastic diseases (*n* = 3, 6.3% from the available data) were identified in a minority of cases [93,94,116] (Supplementary Table S1).

Details on medication exposure known to potentially precipitate or exacerbate MG before diagnosis were available for 27 patients (56.3%). Among patients with available data, nine (33.3%) had received at least one medication potentially associated with MG exacerbation [81,95,98,99,101,105,108,116,121]. Reported agents included glucocorticoids, atorvastatin, azithromycin, unspecified antibiotics, and magnesium supplements. Several of these medications have been previously associated with exacerbation of MG rather than de novo disease induction.

### 3.3. Characteristics of SARS-CoV-2 Infection and Management

Among the included patients, COVID-19 vaccination status was reported in 26 cases (54.2%). In this group, only four patients (15.4%) were vaccinated against COVID-19 before the onset of MG [80,82,89,91]. The method of SARS-CoV-2 diagnosis confirmation was available in 28 patients (58.3%, Supplementary Table S1). The diagnosis was based on RT-PCR (nasopharyngeal swab, *n* = 15, 53.6%), on a combination of RT-PCR and chest CT (*n* = 7, 25.0%), and on serological testing (*n* = 3, 10.7%, Supplementary Table S1). Information on the severity of previous COVID-19 infection was available for 33 (68.8%) of the evaluated patients. Mild disease was the most frequently reported, being observed in 19 patients (57.6%), followed by severe disease in 11 patients (33.3%), and moderate disease in 3 patients (9.1%).

The treatment for COVID-19 infection was variably reported across the included cases. Among patients with available data on glucocorticoid therapy (*n* = 25, 52.1%), 8 patients (32.0%) received glucocorticoids as part of COVID-19 management, dexamethasone being the most frequently reported (*n* = 4), whereas 17 patients (68.0%) did not receive glucocorticoid therapy. Data for antiviral or antibiotic therapy (*n* = 24, 50.0%), of these 13 patients (54.2%) received antibiotic or antiviral therapy. The most frequently used agent was remdesivir (*n* = 7), followed by azithromycin (*n* = 1) and monoclonal antibody therapy (*n* = 1). Combination therapies were also reported and included hydroxychloroquine with azithromycin (*n* = 1) and lopinavir/ritonavir with hydroxychloroquine (*n* = 1).

### 3.4. Findings Related to MG Following SARS-CoV-2 Infection

#### 3.4.1. Clinical Characteristics of MG

The time interval between the onset or diagnosis of COVID-19 and the development of MG was reported for 37 patients (77.1%). This interval ranged from 5 to 300 days, with a median of 21 days (mean 40 ± 59.2 days), indicating substantial variability in temporal association. Of the patients evaluated, 24 cases (50.0%) had available data reporting the number of days between the initial symptoms of MG and the formal diagnosis. The reported interval ranged from 2 to 210 days; the median time was 10 days (IQR 5–44 days, mean 31.8 ± 52.1 days), suggesting potential diagnostic delays or more severe clinical outcomes in a subset of cases.

The initial clinical presentation of MG was available for 47 patients (97.9%). Generalized onset was observed in 37 patients (78.7%), and ocular MG was noted in 10 patients (21.3%) [85,88,92,96,105,107,109,110,113,116]. Among patients with generalized disease, 8 cases (21.6%) had oculo-bulbar involvement, while 2 patients (5.4%) presented predominantly bulbar symptoms at onset. Notably, in all these cases (*n* = 10, 21.3%), no limb involvement was present at disease onset.

Regarding the clinical evolution of MG, data were available for 46 patients (95.9%). Generalized MG remained the predominant form during disease evolution, observed in 36 patients (78.3%). Among patients with ocular onset, all cases remained ocular throughout follow-up.

Information on MGFA (Myasthenia Gravis Foundation of America) classification at onset was available for 18 patients (37.5%). When not explicitly reported, MGFA class I was assigned only in cases with clearly described purely ocular involvement, while no further classification was inferred. MGFA class I was the most frequent presentation, noted in 10 patients (55.6%), MGFA class IIb was reported in 4 patients (22.2%), while MGFA class IIa, IIIa, IIIb, and IVb were each reported in one patient (5.6% each), reflecting a broad spectrum of initial disease severity.

Baseline severity scores were reported in 10 cases (20.8%), multiple scoring systems were used across the studies: Myasthenia Gravis Composite score (MGC, *n* = 4), Myasthenia Gravis Composite Scale (MGCS, *n* = 3), Myasthenia Gravis Activities of Daily Living (MG-ADL, *n* = 1), Quantitative Myasthenia Gravis score (QMG, *n* = 1), and the Besinger score (*n* = 1). Given the variability of scoring systems and the limited number of reported cases, a standardized and objective comparison of baseline disease severity across patients was not feasible.

#### 3.4.2. MG Diagnostic Findings

Antibody status was available for 45 cases (93.8%). AChR-Abs were the most frequently detected (*n* = 30, 66.7%), followed by MuSK-Abs (*n* = 4, 8.9%), and LRP4-Abs (*n* = 2, 4.5%), while five patients were reported as seronegative (Table 4). Among these, one patient had negative results for AChR-Abs, MuSK-Abs, and LRP4-Abs, two patients were described as seronegative without specification of the tested antibodies, one patient had negative AChR-Abs and MuSK-Abs, and one patient had isolated negative AChR-Abs (Table 4). Double seropositivity was reported in three patients as follows: AChR and MuSK antibodies (*n* = 1), AChR and anti-titin antibodies (*n* = 1), and AChR associated with anti-striational antibodies (*n* = 1) (Table 4). Additionally, one patient was described as seropositive without specification of the antibody subtype. Antibody titers were inconsistently reported across studies (Table 4).

Single-fiber electromyography data were reported in 14 patients (29.2%). The test was performed in 8 patients (57.1%). Of these, 7 cases (87.5%) had abnormal findings, while one had a negative result. Data regarding repetitive nerve stimulation were documented in 28 of the 48 patients (58.3%). The test was not performed in 3 patients (10.7%), a decremental response consistent with impaired neuromuscular transmission was observed in 21 cases (75.0%), while negative results were noted in 4 patients (14.3%) (Table 4).

Chest CT imaging data, including pulmonary involvement and thymic status, were available for 36 patients (75.0%). Among these, chest CT was not performed in three patients (8.4%). Of the remaining patients who underwent chest CT, evidence of pneumonia was reported in 14 patients (38.9%), absence of thymic pathology was observed in 25 patients (69.4%), and an anterior mediastinal mass suggestive of thymoma was observed in 6 patients (16.7%). Histopathological confirmation of thymic pathology was noted in three cases and included thymic hyperplasia (*n* = 1), spindle cell thymoma (*n* = 1) and WHO Grade B1 thymoma with abundant CD3^+^ T-cells thymocytes (*n* = 1). Of these, in one case, thymic pathology was confirmed on both chest CT and biopsy. Another patient had been diagnosed with a benign thymoma six years before the MG onset (Table 4).

Thus, thymic pathology was confirmed in nine patients. Among seven patients (77.8%) who were positive for AChR-Abs, one patient was reported as seropositive without specification of the antibody subtype, while in another case, the presence of MG-specific antibodies was not reported. Thus, thymic pathology was identified in 30.0% of AChR-Abs positive patients (Table 4).

Additional diagnostic methods for MG were available for 37 patients (77.0%). Of these, 12 patients (32.4%) underwent at least one additional diagnostic test with positive results: The ice pack test was the most frequently reported method (*n* = 5), either performed alone or in combination with the pyridostigmine test (*n* = 2) or the neostigmine test (*n* = 1). Also, other diagnostic methods included the neostigmine test, Cogan’s lid twitch sign, the Simpson test, edrophonium chloride testing, fatigue testing, and the Wartenberg test (Table 4).

#### 3.4.3. MG Treatment Data

Details on the use of acetylcholinesterase inhibitors (AChEi) were available for 43 cases (89.6%). Among these, AChEi were used in 36 patients (83.7%). Pyridostigmine was the most frequently prescribed AChEi (*n* = 34), followed by neostigmine (*n* = 1) and rivastigmine (*n* = 1).

When specified, the initial pyridostigmine dose ranged from 30 mg to 540 mg/day. Data on glucocorticoid use as initial treatment for MG were available for 44 patients (91.7%), with glucocorticoids administered in 30 cases (68.2%). Regarding glucocorticoid therapy at discharge, data were available for 28 cases (58.3%), of whom 14 (50.0%) were discharged on this treatment.

Data on intravenous immunoglobulin (IVIG) administration were available for 45/48 patients (93.8%). Of these, 21/45 patients (46.7%) were treated with IVIG. Data regarding the use of plasmapheresis were available for 43 cases (89.6%), and plasmapheresis was required in nine patients (20.9%).

Information on the use of immunosuppressive therapy, including azathioprine (AZA), mycophenolate mofetil (MMF), and other agents, was available for 41 patients (85.4%). Among patients with available data, 11 (26.8%) were treated with immunosuppressives. AZA was the most frequently reported agent (*n* = 6), followed by MMF (*n* = 2) and tacrolimus (*n* = 2). One patient was initially treated with tacrolimus, which was subsequently changed to cyclophosphamide after 4 weeks. Another patient was reported as receiving long-term immunosuppression without further specification.

Findings on additional treatment methods were available for 46 patients (95.8%). Of these, additional interventions were applied in 13 cases (28.3%). The most frequently reported interventions included tracheostomy (*n* = 5; 10.9%) and thymectomy (*n* = 4, 8.7%). Other interventions, including the CRISIS management approach, efgartigimod infusions, antibiotic therapy, and hydroxychloroquine, were each reported in single cases.

Adverse events were infrequently reported, which may reflect underreporting rather than a true low incidence, being available in 5 cases (10.4%). Reported adverse events were mainly associated with pyridostigmine, including suboptimal clinical response and intolerance, each observed in two patients. Additionally, one patient developed gastrointestinal side effects, including nausea and diarrhea, associated with the treatment with tacrolimus.

#### 3.4.4. Severe Clinical Course and Respiratory Failure: Interplay Between COVID-19 and MG-Related Complications

A substantial proportion of patients required intensive care unit (ICU) admission, suggesting a severe clinical course. Data on ICU admission were available for 42 cases (87.5%), among whom 20 (47.6%) required ICU care.

Information on the development of myasthenic crisis was available for 39 individuals (81.3%). Of these, the crisis was observed in 17 cases (43.6%).

Respiratory failure was common, often related to MG or an overlap effect of both MG and SARS-CoV-2 infection. Data were available for 44 patients (91.7%), and respiratory failure was reported in 20 cases (45.5%). Among these patients, 11 cases (55.0%) were attributed to MG, while 9 cases (45.0%) were considered to have an overlapping contribution. Notably, one patient developed respiratory failure secondary to a myasthenic crisis occurring two months after diagnosis.

Findings on the need for mechanical ventilation were available for 43 patients (89.6%). Of these, 17 patients (39.5%) required mechanical ventilation, including one patient who required several episodes of ventilatory support.

Data on other associated infections were available for 32 cases (66.7%), of whom two cases (6.3%) were reported as having additional infections.

### 3.5. Outcome

Clinical outcomes were available for 42 patients (87.5%). Most patients showed clinical improvement (*n* = 33, 78.6%); complete recovery was observed in five cases (11.9%), spontaneous recovery in two patients (4.8%), and a stable disease course in one patient (2.4%). One patient died one year after MG diagnosis due to multiorgan failure unrelated to the disease. Of the patients with spontaneous recovery, one received no treatment, and another was treated with pyridostigmine 180 mg/day. Reporting of follow-up outcomes using standardized MG severity scales was notably limited, with only six cases (12.5%) available. Among the reported cases, different scoring systems were used, including MG-ADL (*n* = 1), QMG (*n* = 1), Besinger score (*n* = 1), and MGC (*n* = 3). This variability limits direct comparisons across cases, reflects the absence of standardized reporting practices in the current literature, and highlights an important gap in the available evidence.

Information on residual disability was available for 18 cases (37.5%). Among patients with available data, 9 patients (50.0%) reported residual disability, including ocular involvement (*n* = 4, 44.4%) and limb weakness (*n* = 4, 44.4%), followed by dysphagia, dysarthria, and chewing fatigability (each *n* = 1). Some patients exhibited more than one residual deficit.

## 4. Discussion

The currently available studies regarding new-onset MG following SARS-CoV-2 infection were synthesized in this SLR, including 44 studies describing 48 patients, the majority being single-patient CRs. The findings suggest that MG developing after COVID-19 typically occurs within a relatively short latency period, with a median interval of 21 days. Most patients presented with generalized onset and were predominantly positive for AChR-Abs, consistent with the established clinical and serological profile of classical MG, in which AChR-Abs represent the most common subtype, particularly in generalized disease [13,14,127]. MuSK- and LRP4-positive cases were less frequent, paralleling the known distribution of antibody subtypes in conventional MG [123,128,129].

Despite similarities in phenotype, a substantial proportion of patients had severe clinical courses, with nearly half requiring ICU admission and more than one-third requiring mechanical ventilation. These findings underscore the potential clinical impact of this association. However, most patients showed clinical improvement following conventional MG therapies, suggesting preserved treatment responsiveness comparable to classical MG [14,130].

The coexistence of MG with other ADs has been reported widely in the literature [131,132]. In this SLR, personal history of ADs was identified in 13.2% of patients with available data, while a family history of ADs was rarely reported, being documented in only two cases. The associated ADs observed included Graves’ disease, Hashimoto’s thyroiditis, GAD-65 seropositive multifocal encephalitis, antiphospholipid syndrome, psoriasis, and autoimmune gastritis. These findings are broadly consistent with larger epidemiological and cohort-based studies, which have identified autoimmune thyroid disease as the most frequent comorbidity in MG, followed by other systemic and neurological autoimmune diseases [131,133]. In a cohort of 796 patients with MG, ADs were reported in 11.6% of cases, with thyroid disorders being the most prevalent. These observations support the concept of a shared autoimmune predisposition in at least a subset of patients with MG.

The wide range of latency intervals observed in this review may reflect the complexity of the underlying pathophysiological mechanisms. SARS-CoV-2 infection may act either as an immunological trigger or as a factor unmasking pre-existing subclinical MG in susceptible individuals [20]. This interpretation is supported by the observation that the clinical phenotype, antibody profile, and thymic abnormalities identified in several patients closely resemble those observed in classical MG, particularly in AChR-positive disease [14,130,134]. Although the temporal relationship may support a possible post-infectious autoimmune phenomenon, a coincidental association cannot be excluded, especially given the high global prevalence of COVID-19 [20].

Our findings also suggest that post-COVID MG may present with a severe clinical course in a considerable proportion of reported cases. Nearly half of the patients required ICU admission, more than 40% developed myasthenic crisis, and a substantial proportion required mechanical ventilation. These proportions appear higher than those typically reported in large MG cohorts, in which myasthenic crisis and respiratory failure occur in a smaller subset of patients over the disease course [13,14,19,130,135]. However, these observations should be interpreted with caution, as the available evidence is largely derived from CR and small CS, which are inherently subject to publication bias, with more severe or unusual cases being preferent ially reported, potentially leading to an overestimation of the severity of MG occurring after SARS-CoV-2 infection in the current literature.

Respiratory impairment in these patients may not be solely attributable to MG. SARS-CoV-2 infection itself can lead to pulmonary involvement, systemic inflammation, and neuromuscular dysfunction, all of which may contribute to respiratory compromise independent of neuromuscular junction failure [6,8,9,48]. In several cases included in this review, respiratory failure was described as having overlapping contributions from both MG and COVID-19, further complicating direct comparisons with conventional MG cohorts.

Despite the severity observed in some patients, the overall therapeutic response appears favorable. Most patients responded to standard MG therapies, including AChEi, glucocorticoids, intravenous immunoglobulin, and plasmapheresis. This suggests that MG developing after SARS-CoV-2 infection does not differ substantially from classical MG in terms of treatment responsiveness, and that established management strategies remain appropriate [13,14,16,20,130].

However, conclusions regarding prognosis should be interpreted cautiously. Baseline disease severity and follow-up outcomes were reported using different assessment instruments, including MG-ADL, QMG, MGC, MGCS, and the Besinger score. In addition, outcome reporting was available for only a limited number of patients. This heterogeneity precludes meaningful quantitative comparisons across studies and limits the ability to draw firm conclusions regarding the long-term prognosis of MG occurring after SARS-CoV-2 infection.

At present, epidemiological evidence supporting a true increase in MG incidence following COVID-19 remains limited and inconclusive [11,73]. While some observational studies and real-world data analyses suggest a possible association, robust population-level data are lacking [11,16,44,65,66,67,73]. Therefore, current evidence supports the biological plausibility of SARS-CoV-2 as a potential immune trigger or disease-unmasking factor, but remains insufficient to establish a definitive causal relationship [20].

Several limitations should be considered when interpreting the findings of this review. First, the available evidence is predominantly derived from CR and small CS, increasing susceptibility to publication and reporting bias. Severe, unusual, or clinically dramatic presentations are more likely to be recognized, reported, and published than mild or self-limited cases. Consequently, the high rates of ICU admission, myasthenic crisis, respiratory failure, and mechanical ventilation observed in this review may overestimate the true severity of MG occurring after SARS-CoV-2 infection. Second, substantial variability existed across the included studies regarding diagnostic evaluation and reporting. Diagnostic confirmation was not standardized, and several studies lacked complete information on antibody testing, electrophysiological assessment, MGFA classification, thymic imaging or histopathological evaluation, and long-term outcomes. This heterogeneity may have influenced case classification, limited the comparability of patients across studies, and affected the interpretation of the findings. Finally, given the high global prevalence of COVID-19, some temporal associations may be coincidental. Moreover, the possibility that SARS-CoV-2 infection may unmask pre-existing subclinical MG rather than induce a truly new autoimmune disorder cannot be excluded.

Future research should focus on multicentre cohort studies and comparative epidemiological analyses to better define the incidence and clinical characteristics of MG following SARS-CoV-2 infection. Prospective studies incorporating standardized diagnostic criteria, antibody panels, electrophysiological testing, and validated outcome measures are needed. In parallel, mechanistic studies exploring molecular mimicry, immune dysregulation, and thymic involvement may provide further insight into the potential link between SARS-CoV-2 infection and MG pathogenesis. Third, clinical severity and outcome measures were inconsistently reported across studies. Different assessment instruments were used, including MG-ADL, QMG, MGC, MGCS, and the Besinger score, while standardized follow-up data were available for only a limited number of patients. As a result, direct comparisons between cases were not feasible, and conclusions regarding prognosis should be interpreted with caution.

## 5. Conclusions

Reported cases of MG occurring after developing SARS-CoV-2 infection appear to be a rare but clinically relevant condition occurring within weeks after infection, typically presenting as a generalized form, and is most frequently associated with AChR-Abs positivity, closely resembling classical MG phenotypes [14,130].

Although severe clinical evolution, including ICU admission, myasthenic crisis, and respiratory failure, were frequently reported, these findings are likely influenced by publication bias and by the overlapping respiratory burden of COVID-19 itself. Importantly, most patients responded well to conventional MG therapies, suggesting similar treatment responsiveness to classical MG [13,14,130].

The currently available evidence may suggest a possible post-infectious autoimmune phenomenon; however, causality cannot be established based on the currently available evidence. SARS-CoV-2 may act as an immunological trigger or as a factor that unmasks latent autoimmunity in susceptible individuals, but further studies are required to clarify this relationship [20].

Overall, clinicians should remain aware of the possibility of MG in patients presenting with compatible symptoms following COVID-19, as early recognition and appropriate treatment may significantly improve outcomes.

## Figures and Tables

**Table 1:** Search strategies (database^®^: search string).

**PubMed**^®^: #1 Myasthenia Gravis [MeSH Terms] #2 “myasthenia gravis”[title/abstract] #3 (#1) OR (#2) #4 COVID-19 OR SARS-CoV-2 [MeSH Terms] #5 covid*[Title/Abstract] OR “SARS-CoV-2”[Title/Abstract] OR coronavirus[Title/Abstract] #6 (#4) OR (#5) #7 “new-onset”[Title/Abstract] OR “de novo”[Title/Abstract] OR “first presentation”[Title/Abstract] OR “initial presentation”[Title/Abstract] Broad search: #8 (#3) AND (#6) Filters: English, from 2019–2025 Focused search: #9 (#3) AND (#6) AND (#7) Filters: English, from 2019–2025
**Ovid Embase**^®^: 1. ‘myasthenia gravis’/exp 2. (myasthenia gravis).ti,ab. 3. 1 or 2 4. ‘covid-19’/exp OR ‘SARS-CoV-2’/exp 5. (covid* or “sars-cov-2” or coronavirus).ti,ab. 6. 4 or 5 7. (new-onset or de novo or “first presentation” or “initial presentation”).ti,ab. Broad search: 8. 3 and 6 Filters: English, from 2019–2025 Focused search: 9. 3 and 6 and 7 Filters: English, from 2019–2025
**Ovid^®^ Medline**: 1 exp myasthenia gravis/ 2 myasthenia gravis.ti,ab. 3 1 or 2 4 exp COVID-19/or SARS-CoV-2/ 5 (covid* or sars-cov-2 or coronavirus).ti,ab. 6 4 or 5 7 (new-onset or de novo or “first presentation” or “initial presentation”).ti,ab. Broad search: 8 3 and 6 Filters: English, from 2019–2025 Focused search: 9 3 and 6 and 7 Filters: English, from 2019–2025
**Scopus**^®^: Broad search: TITLE-ABS-KEY(“myasthenia gravis”) AND TITLE-ABS-KEY (covid* OR “sars-cov-2” OR coronavirus) AND LANGUAGE (“English”) AND (PUBYEAR > 2018 AND PUBYEAR < 2026) Focused search: TITLE-ABS-KEY(“myasthenia gravis”) AND TITLE-ABS-KEY (covid* OR “sars-cov-2” OR coronavirus) AND TITLE-ABS-KEY (“new-onset” OR “de novo” OR “first presentation” OR “initial presentation”) LANGUAGE (“English”) AND (PUBYEAR > 2018 AND PUBYEAR < 2026)
**WOS**^®^: Broad: TS = (“myasthenia gravis”) AND TS = (covid* OR “sars-cov-2” OR coronavirus) AND PY = (2019–2025) AND LA = (“English”) Focused: TS = (“myasthenia gravis”) AND TS = (covid* OR “sars-cov-2” OR coronavirus) AND TS = (“new-onset” OR “de novo” OR “first presentation” OR “initial presentation”) AND PY = (2019–2025) AND LA = (“English”)

* Truncation wildcard used to retrieve word variants.

**Table 2:** Excluded reports and reasons.

Reason for Exclusion	Database Studies	Database Reviews
language: non-English	[23,36,38,68,124]	[62]
type: letter to the editor referring to already published studies		[28,42,125]
type: review without new reported cases		[12,16,17,20,23,24,26,29,31,32,34,43,44,45,47,48,49,50,52,57,58,59,69,70,71,72,126]
data: pediatric population	[25,61]	
data: overlapping from the same study	[55]	
design: non-MG diagnosis	[33,35,39,46,51,56,60]	[78]
design: non-SARS-CoV-2 diagnosis	[40,53,77,126]	
design: MG onset/diagnosis before SARS-CoV-2 diagnosis	[30,37,41,54,75,76]	
design: vaccines-related		[63]
Design: epidemiological studies without time correlation between MG and SARS-CoV-2 infection established	[11,64,65,66,67,73,74]	

**Table 3:** Characteristics of included studies (*n* = 44).

Citation	Type	Year	Country	Design	Presentation Year	JBI/JBICS
Abdullah et al. [80]	MA	2023	USA	CR	NR	1
Alboini et al. [81]	MA	2022	Italy	CR	NR	6
Ali et al. [82]	MA	2021	USA	CR	NR	2
Mahmoud et al. [83]	MA	2022	USA	CR	NR	4
Annabi-Rabadi et al. [84]	MA	2023	USA	CR	NR	1
Assini et al. [85]	JA	2021	Italy	CR	2020	6
Banerjee et al. [86]	JA	2023	India	CR	NR	2
Barroso et al. [87]	MA	2022	Colombia	CR	NR	0
Bhandarwar et al. [88]	JA	2021	India	CR	2020	4
Brossard-Barbosa et al. [89]	JA	2022	Canada	CR	NR	6
Castro Silva et al. [90]	JA	2024	Portugal	CR	2022	4
Chatterjee et al. [91]	JA	2022	USA	CR	2020	6
Croitoru et al. [92]	JA	2022	Romania	CR	2021	7
De Giglio et al. [93]	JA	2022	Italy	CR	2020	6
Feiz et al. [94]	JA	2024	USA	CR	NR	6
Gigilashvili et al. [95]	MA	2024	USA	CR	NR	2
Hiraoka et al. [96]	JA	2025	Japan	CR	NR	5
Huber et al. [97]	JA	2020	Germany	CR	2020	7
Jha et al. [98]	JA	2024	India	CR	NR	3
Jogy et al. [99]	JA	2022	Estonia	CR	NR	6
Karimi et al. (*n* = 3) [100]	JA	2021	Iran	CS	2020	7
Kepfinger et al. [101]	MA	2024	USA	CR	NR	2
Khairandish et al. [102]	JA	2025	USA	CR	NR	2
Laizane et al. [103]	MA	2024	Latvia	CR	NR	0
Minca et al. [104]	JA	2024	Romania	CR	NR	5
Muhammed et al. [105]	JA	2021	United Kingdom	CR	2020	5
Nakano et al. [106]	JA	2025	Japan	CR	NR	6
Portugal et al. [107]	MA	2022	Mexico	CR	NR	0
Perez Alvarez et al. [108]	JA	2020	Spain	CR	NR	6
Perry et al. [109]	MA	2022	USA	CR	NR	1
Popescu et al. [110]	JA	2023	France	CR	2023	5
Rahimian et al. [111]	JA	2022	Iran	CR	2020	4
Reddy et al. [112]	JA	2021	India	CR	2020	6
Restivo et al. (*n* = 3) [113]	JA	2020	Italy	CS	2020	6
Rogers et al. [114]	MA	2023	USA	CR	NR	1
Sadiq et al. [115]	JA	2023	USA	CR	NR	4
Sittol et al. [116]	MA	2020	USA	CR	NR	2
Sriwastawa et al. [117]	JA	2020	USA	CR	NR	5
Syed et al. [118]	MA	2022	USA	CR	NR	1
Taheri et al. [119]	JA	2022	Iran	CR	2021	6
Tereshko et al. [120]	JA	2022	Italy	CR	2021	6
Tugasworo et al. [121]	JA	2021	Indonesia	CR	NR	3
Valjarevic et al. [122]	JA	2023	Serbia	CR	2021	4
Wong et al. [127]	MA	2022	USA	CR	NR	1

Abbreviations: CR—case report, CS—case series, JA—journal article, JBI/JBICS—Joanna Briggs Institute critical appraisal checklist for case reports/case series, MA-meeting abstract, NR-not reported.

**Table 4:** Summary of diagnostic findings in patients with new-onset MG following SARS-CoV-2 infection (*n* = 48).

Diagnostic Variable	Patients with Available Data, *n* (%)	Positive/Abnormal Findings, *n* (%)
AChR antibodies	45 (93.8)	30 (66.7)
MuSK antibodies	45 (93.8)	4 (8.9)
LRP4 antibodies	45 (93.8)	2 (4.5)
Seronegative MG	45 (93.8)	5 (11.1)
Double seropositivity	45 (93.8)	3 (6.7)
SFEMG performed	14 (29.2)	7/8 abnormal (87.5% of tested patients)
RNS performed	28 (58.3)	21 (75.0)
Additional diagnostic tests performed *	37 (77.0)	12 (32.4)
Chest CT performed	36 (75.0)	14 pneumonia (38.9)
Thymic pathology on imaging and/or histopathology	36 (75.0)	9 (25.0)
MGFA classification reported	18 (37.5)	N/A
MGFA class I	18 (37.5)	10 (55.6)
MGFA class IIa	18 (37.5)	1 (5.6)
MGFA class IIb	18 (37.5)	4 (22.2)
MGFA class IIIa	18 (37.5)	1 (5.6)
MGFA class IIIb	18 (37.5)	1 (5.6)
MGFA class IVb	18 (37.5)	1 (5.6)

* Additional diagnostic tests included ice-pack test, neostigmine test, pyridostigmine test, Cogan’s lid twitch sign, Simpson test, edrophonium chloride test, fatigue testing, and Wartenberg test. Abbreviations: AChR = acetylcholine receptor; MuSK = muscle-specific kinase; LRP4 = low-density lipoprotein receptor-related protein 4; SFEMG = single-fibre electromyography; RNS = repetitive nerve stimulation; MGFA = Myasthenia Gravis Foundation of America.

## Data Availability

Data extracted from published studies are contained within the article and Supplementary Materials. Further details are available from the corresponding author upon reasonable request.

## Supplementary Materials

The following supporting information can be downloaded at: https://www.mdpi.com/article/10.3390/life16071100/s1. Table S1. Clinical characteristics of patients with new-onset myasthenia gravis following SARS-CoV-2 infection (*n* = 48); Table S2. PRISMA 2020 checklist.

## Abbreviations

The following abbreviations are used in this manuscript:

AChE inhibitors	acetylcholinesterase inhibitors
AChR-abs	acetylcholine receptor antibodies
AChR	acetylcholine receptor
ADs	autoimmune diseases
COVID-19	Coronavirus Disease 2019
CR	case report
CS	case series
WHO	World Health Organisation
MG	myasthenia gravis
MuSK-abs	muscle-specific kinase antibodies
LRP4-Abs	low-density lipoprotein receptor-related protein 4 antibodies
SARS-CoV-2	severe acute respiratory syndrome coronavirus 2
