# Supplementary material for: New-Onset Myasthenia Gravis Associated with SARS-CoV-2 Infection: A Systematic Review
Source: Life (Basel). 2026 Jun 30;16(7):1100. doi: 10.3390/life16071100 (PMC13412210; doi:10.3390/life16071100)
Supplement: Supplementary file 1 [file life-16-01100-s001.zip › Table S1. Clinical characteristics of patients with new-onset myasthenia gravis following SARS-CoV-2 infection (n=48).pdf]

Supplementary Table S1. Clinical characteristics of patients with new-onset myasthenia gravis following SARS-CoV-2 infection (n=48)

citation	sex	age (y)	Autoimmune comorbidities	Neurological comorbidities	Vaccination status	COVID-19 severity	COVID-19–MG interval (d)	MG symptom-to-diagnosis interval (d)	MG at onset (ocular/generalized)	MG in evolution (ocular/generalized)	Antibody status	RNS	SFEMG	Chest imaging (lung/thymus)	Thymic pathology	Myasthenic crisis	Need for ICU admission	Respiratory failure	Mechanical ventilation	AChE inhibitors (pyridostigmine/ neostigmine/rivastigmine)	Clinical outcome
Abdullah et al [98]	F	68	none	none	NR	NR	21	NR	G	G	seronegative (unspecified antibodies tested)	NR	NR	NR	NR	Y	Y	Y (MG-related)	Y	N	improved
Alboini et al [99]	F	20	none	none	Y	NR	30	NR	G	G	MuSK-Abs	decremental response	NR	NR	NR	Y	Y	Y (MG-related)	Y	pyridostigmine	improved
Ali et al [100]	F	42	GAD-65 seropositive multifocal encephalitis	GAD-65 seropositive multifocal encephalitis	N	NR	NR	NR	G	G	AChR-Abs	decremental response	NR	hyperintensity consistent with thymoma	Chest-MRI-hyperintensity consistent with thymoma, WHO Grade B1 thymoma with abundant CD3+	Y	Y	Y (MG-related)	Y	NR	improved

citation	sex	age (y)	Autoimmune comorbidities	Neurological comorbidities	Vaccination status	COVID-19 severity	COVID-19–MG interval (d)	MG symptom-to-diagnosis interval (d)	MG at onset (ocular/generalized)	MG in evolution (ocular/generalized)	Antibody status	RNS	SFEMG	Chest imaging (lung/thymus)	Thymic pathology	Myasthenic crisis	Need for ICU admission	Respiratory failure	Mechanical ventilation	AChE inhibitors (pyridostigmine/neostigmine/rivastigmine)	Clinical outcome
															T-cells thymocytes (biopsy)						
Anas et al [101]	M	67	none	none	Y	mild	NR	17	G	G	AChR-Abs	NR	NR	no thymic pathology, no pneumonia	negative chest CT scan	N	Y	N	N	pyridostigmine	improved
Annabi-Rabadi et al [102]	F	90	none	none	NR	severe	7	NR	G (predominantly bulbar)	G	AChR-Abs	NR	NR	anterior mediastinal mass suggestive of thymoma	yes, imaging	Y	NR	Y (overlap)	NR	NR	improved
Assini et al [30]	M	77	none	none	N	severe	56	NR	G (oculo-bulbar)	G	MuSK-Abs	decremental response	positive	no thymic pathology, pneumonia positi	negative chest CT scan	N	N	N	N	pyridostigmine	improved

citation	sex	age (y)	Autoimmune comorbidities	Neurological comorbidities	Vaccination status	COVID-19 severity	COVID-19–MG interval (d)	MG symptom-to-diagnosis interval (d)	MG at onset (ocular/generalized)	MG in evolution (ocular/generalized)	Antibody status	RNS	SFEMG	Chest imaging (lung/thymus)	Thymic pathology	Myasthenic crisis	Need for ICU admission	Respiratory failure	Mechanical ventilation	AChE inhibitors (pyridostigmine/neostigmine/rivastigmine)	Clinical outcome
														ve 42 days before							
Banerjee et al [103]	M	47	NR	NR	N	mild	43	10	O	O	AChR-Abs	decremental response	NR	no thymic pathology, no pneumonia	negative chest CT scan	N	N	N	N	pyridostigmine	improved
Barroso et al [104]	NR	NR	NR	NR	NR	NR	NR	NR	G	NR	NR	NR	NR	NR	NR	NR	NR	NR	NR	NR	NR
Bhandarwar et al [105]	M	61	none	none	N	mild	60	NR	G	G	AChR-Abs	NR	NR	anterior mediastinum mass suggestive of thymoma	spindle cell thymoma (biopsy)	N	NR	Y (MG-related)	N	pyridostigmine	improved
Brossard-Barbosa el al [106]	M	60	none	none	NR	mild	30	6	O	O	AChR-Abs	unperformed	unperformed	unperformed	NR	N	N	N	N	pyridostigmine	improved

citation	sex	age (y)	Autoimmune comorbidities	Neurological comorbidities	Vaccination status	COVID-19 severity	COVID-19–MG interval (d)	MG symptom-to-diagnosis interval (d)	MG at onset (ocular/generalized)	MG in evolution (ocular/generalized)	Antibody status	RNS	SFEMG	Chest imaging (lung/thymus)	Thymic pathology	Myasthenic crisis	Need for ICU admission	Respiratory failure	Mechanical ventilation	AChE inhibitors (pyridostigmine/ neostigmine/rivastigmine)	Clinical outcome
Castro Silva et al [107]	F	78	none	none	Y	mild	14	5	G	G	seronegative (AChR-Abs, MuSK-Abs, LRP4-Abs)	negative	negative	no thymic pathology	negative chest CT scan	Y	Y	Y (MG-related)	N	pyridostigmine	improved
Chatterjee et al [108]	M	83	none	trigeminal neuralgia	N	severe	47	210	G	G	AChR-Abs	NR	NR	no thymic pathology, pneumonia positive	negative chest CT scan	Y	Y	Y (MG-related)	Y	pyridostigmine	Death (1 year later; multiorgan failure, non-MG-related)
Croitoru et al [109]	M	78	none	NR	Y	mild	9	4	G (oculo-bulbar)	G	AChR-Abs	decremental response	unperformed	no thymic pathology	negative chest CT scan	Y	Y	Y (MG-related 2 months later)	Y	pyridostigmine	improved
De Giglio et	M	75	Graves disease	NR	N	NR	63	7	O	O	AChR-Abs	decremental	positive	no thymic	negative chest	N	N	N	N	pyridostigmine	complete recovery

citation	sex	age (y)	Autoimmune comorbidities	Neurological comorbidities	Vaccination status	COVID-19 severity	COVID-19–MG interval (d)	MG symptom-to-diagnosis interval (d)	MG at onset (ocular/generalized)	MG in evolution (ocular/generalized)	Antibody status	RNS	SFEMG	Chest imaging (lung/thymus)	Thymic pathology	Myasthenic crisis	Need for ICU admission	Respiratory failure	Mechanical ventilation	AChE inhibitors (pyridostigmine/ neostigmine/rivastigmine)	Clinical outcome
al [110]												response		pathology	CT scan						
Feiz et al [111]	M	81	none	none	NR	NR	120	NR	G	G	AChR-Abs	NR	NR	no thymic pathology, no pneumonia	NR	Y	Y	Y (MG-related)	Y	pyridostigmine	complete recovery
Gigilashvili et al [112]	F	36	NR	NR	NR	NR	14	NR	G	G	AChR-Abs	NR	NR	NR	NR	NR	N	N	N	pyridostigmine	complete recovery
Hiraoka et al [113]	F	78	none	right hemiplegia secondary to cerebral infarction	NR	NR	7	90	G	G	double seropositive (AChR-Abs , MuSK-Abs)	decremental response	unperformed	no thymic pathology	negative chest CT scan	N	N	N	N	N	improved
Huber et al [114]	F	21	none	none	N	mild	21	5	O	O	AChR-Abs	negative	NR	no thymic pathology, no pneumonia	negative thoracic MRI	N	N	N	N	pyridostigmine	improved

citation	sex	age (y)	Autoimmune comorbidities	Neurological comorbidities	Vaccination status	COVID-19 severity	COVID-19–MG interval (d)	MG symptom-to-diagnosis interval (d)	MG at onset (ocular/generalized)	MG in evolution (ocular/generalized)	Antibody status	RNS	SFEMG	Chest imaging (lung/thymus)	Thymic pathology	Myasthenic crisis	Need for ICU admission	Respiratory failure	Mechanical ventilation	AChE inhibitors (pyridostigmine/neostigmine/rivastigmine)	Clinical outcome
Jha et al [115]	F	38	NR	NR	NR	severe	7	30	G (oculo-bulbar)	G	MuSK-Abs	decremental response	NR	no thymic pathology	negative chest CT scan	N	N	N	N	pyridostigmine	complete recovery
Jogy et al [116]	M	65	none	none	N	mild	14	58	G (oculo-bulbar)	G	double seropositive (AChR-Abs, anti-titin-Abs)	decremental response	unperformed	no thymic pathology	negative chest CT scan	N	N	N	N	pyridostigmine	improved
Karimi et al (a) [117]	F	61	none	none	N	moderate	32	10	G	G	AChR-Abs	decremental response	NR	anterior mediastinal mass suggestive of thymoma, pneumonia positive 6 weeks before	yes, imaging	N	NR	NR	N	pyridostigmine	improved
Karimi et al (b) [117]	M	57	none	none	N	mild	6	7	G	G	AChR-Abs	decremental	NR	no thymic pathol	negative chest	N	N	N	N	pyridostigmine	improved

citation	sex	age (y)	Autoimmune comorbidities	Neurological comorbidities	Vaccination status	COVID-19 severity	COVID-19–MG interval (d)	MG symptom-to-diagnosis interval (d)	MG at onset (ocular/generalized)	MG in evolution (ocular/generalized)	Antibody status	RNS	SFEMG	Chest imaging (lung/thymus)	Thymic pathology	Myasthenic crisis	Need for ICU admission	Respiratory failure	Mechanical ventilation	AChE inhibitors (pyridostigmine/neostigmine/rivastigmine)	Clinical outcome
												response		ogy, pneumonia positive 6 days before	CT scan						
Karimi et al (c) [117]	F	38	none	none	N	mild	28	NR	G	G	AChR-Abs	decremental response	NR	no thymic pathology, pneumonia positive	negative chest CT scan	N	N	N	N	pyridostigmine	improved
Kepfinger et al [118]	F	27	NR	NR	NR	severe	NR	NR	G	G	seronegative (unspecified antibodies tested)	NR	NR	NR	NR	Y	Y	Y (overlap)	Y	pyridostigmine	improved
Khairandish et al [119]	F	65	antiphospholipid syndrome	stroke	NR	NR	NR	NR	G	G	LRP4-Abs	NR	NR	no thymic pathology, mild mosaic attenuation in the	NR	N	N	N	N	pyridostigmine	improved

citation	sex	age (y)	Autoimmune comorbidities	Neurological comorbidities	Vaccination status	COVID-19 severity	COVID-19–MG interval (d)	MG symptom-to-diagnosis interval (d)	MG at onset (ocular/generalized)	MG in evolution (ocular/generalized)	Antibody status	RNS	SFEMG	Chest imaging (lung/thymus)	Thymic pathology	Myasthenic crisis	Need for ICU admission	Respiratory failure	Mechanical ventilation	AChE inhibitors (pyridostigmine/neostigmine/rivastigmine)	Clinical outcome
														dependent bilateral lower lobes							
Laizane et al [120]	F	22	NR	NR	NR	moderate	NR	NR	G	G	AChR-Abs	NR	NR	NR	NR	NR	NR	NR	NR	NR	NR
Minca et al [121]	F	30	none	none	N	severe	5	NR	G	G	AChR-Abs	NR	NR	no thymic pathology, pneumonia positive	negative chest CT scan	Y	Y	Y (overlap)	Y	pyridostigmine	improved
Muhammed et al [29]	F	24	none	none	N	mild	28	NR	G	G	MuSK-Abs	decremental response	positive	no thymic pathology	negative chest CT scan	N	Y	N	N	pyridostigmine	stable
Nakano et al [122]	F	46	none	none	NR	mild	NR	14	O	O	seronegative (AChR-Abs, MuSK-Abs)	decremental response	NR	NR	NR	NR	N	N	N	pyridostigmine	improved
Portugal el	M	33	NR	NR	NR	moderate	180	NR	NR	NR	seropositive (unspecif	NR	NR	anterior media	yes, imaging	NR	NR	NR	NR	NR	NR

citation	sex	age (y)	Autoimmune comorbidities	Neurological comorbidities	Vaccination status	COVID-19 severity	COVID-19–MG interval (d)	MG symptom-to-diagnosis interval (d)	MG at onset (ocular/generalized)	MG in evolution (ocular/generalized)	Antibody status	RNS	SFEMG	Chest imaging (lung/thymus)	Thymic pathology	Myasthenic crisis	Need for ICU admission	Respiratory failure	Mechanical ventilation	AChE inhibitors (pyridostigmine/neostigmine/rivastigmine)	Clinical outcome
al [123]											ied antibodies tested)			stinal mass suggestive of thymoma							
Perez Alvarez et al [124]	M	48	psoriasis	none	NR	mild	15	NR	O	O	AChR-Abs	NR	NR	pneumonia positive	NR	N	N	N	NR	N	complete recovery
Perry et al [125]	M	79	NR	NR	NR	severe	32	NR	G	G	AChR-Abs	unperformed	unperformed	no thymic pathology	negative chest CT scan	NR	Y	Y (overlap)	Y (multiple)	rivastigmine	improved
Popescu et al [126]	F	77	none	none	NR	NR	5	NR	O	O	AChR-Abs	negative	NR	no thymic pathology, no pneumonia	negative chest CT scan	N	N	N	N	N	spontaneous recovery after 10 days
Rahimian et al [127]	F	31	none	none	N	NR	NR	4	O	O	seronegative (AChR-Abs)	negative	positive	no thymic pathology, pneumonia	negative chest CT scan	N	N	N	N	pyridostigmine	spontaneous recovery after 14 days

citation	sex	age (y)	Autoimmune comorbidities	Neurological comorbidities	Vaccination status	COVID-19 severity	COVID-19–MG interval (d)	MG symptom-to-diagnosis interval (d)	MG at onset (ocular/generalized)	MG in evolution (ocular/generalized)	Antibody status	RNS	SFEMG	Chest imaging (lung/thymus)	Thymic pathology	Myasthenic crisis	Need for ICU admission	Respiratory failure	Mechanical ventilation	AChE inhibitors (pyridostigmine/neostigmine/rivastigmine)	Clinical outcome
														positive							
Reddy et al [128]	M	65	none	none	N	mild	42	2	G (predominantly bulbar)	G	AChR-Abs	decremental response	NR	no thymic pathology	negative chest CT scan	Y	Y	Y (MG-related)	Y	pyridostigmine	improved
Restivo et al (a) [129]	M	64	none (neurological)	NR	N	mild	5	NR	G	G	AChR-Abs	decremental response	NR	no thymic pathology	negative chest CT scan	NR	N	N	N	pyridostigmine	improved
Restivo et al (b) [129]	M	68	none (neurological)	NR	N	mild	7	NR	G	G	AChR-Abs	decremental response	NR	no thymic pathology	negative chest CT scan	NR	N	N	N	N	improved
Restivo et al (c) [129]	F	71	none (neurological)	NR	N	severe	5	NR	G (oculo-bulbar)	G	AChR-Abs	decremental response	NR	no thymic pathology, pneumonia positive	negative chest CT scan	Y	Y	Y (overlap)	Y	N	improved
Rogers et al [130]	M	78	none	none	NR	NR	28	60	O	O	AChR-Abs	NR	NR	NR	NR	N	N	N	N	pyridostigmine	NR

citation	sex	age (y)	Autoimmune comorbidities	Neurological comorbidities	Vaccination status	COVID-19 severity	COVID-19–MG interval (d)	MG symptom-to-diagnosis interval (d)	MG at onset (ocular/generalized)	MG in evolution (ocular/generalized)	Antibody status	RNS	SFEMG	Chest imaging (lung/thymus)	Thymic pathology	Myasthenic crisis	Need for ICU admission	Respiratory failure	Mechanical ventilation	AChE inhibitors (pyridostigmine/ neostigmine/rivastigmine)	Clinical outcome
Sadiq et al [131]	M	46	none	none	NR	severe	152	NR	G	G	LRP4-Abs	unperformed	unperformed	no thymic pathology, pneumonia positive	negative chest CT scan	Y	Y	Y (overlap)	Y	pyridostigmine	improved
Sittol et al [132]	F	35	none	none	N	severe	6	NR	G	G	AChR-Abs	NR	NR	anterior mediastinum mass suggestive of thymoma, pneumonia positive	yes-imaging	Y	Y	Y (overlap)	Y	pyridostigmine	improved
Sriwastawa et al [133]	F	65	none	meningioma	N	mild	11	3	O	O	double seropositive (AChR-Abs , anti-striational-Abs)	decremental response	NR	no thymic pathology, no pneumonia	negative chest CT scan	N	N	N	N	pyridostigmine	improved

citation	sex	age (y)	Autoimmune comorbidities	Neurological comorbidities	Vaccination status	COVID-19 severity	COVID-19–MG interval (d)	MG symptom-to-diagnosis interval (d)	MG at onset (ocular/generalized)	MG in evolution (ocular/generalized)	Antibody status	RNS	SFEMG	Chest imaging (lung/thymus)	Thymic pathology	Myasthenic crisis	Need for ICU admission	Respiratory failure	Mechanical ventilation	AChE inhibitors (pyridostigmine/neostigmine/rivastigmine)	Clinical outcome
Syed et al [134]	M	72	none	none	NR	severe	NR	NR	G	G	NR	NR	NR	no thymic pathology, pneumonia positive	NR	Y	Y	Y (overlap)	Y	pyridostigmine	NR
Taheri et al [135]	F	35	none	none	NR	mild	17	3	G	G	AChR-Abs	NR	positive	no thymic pathology, pneumonia positive	negative chest CT scan	N	N	N	N	pyridostigmine	improved
Tereshko et al [136]	F	19	Hashimoto's Thyroiditis, autoimmune gastritis	none	N	mild	13	93	G (oculo-bulbar)	G	AChR-Abs	decremental response	positive	unperformed	Thymic Hyperplasia (biopsy)	Y	Y	Y (MG-related)	Y	pyridostigmine	improved

citation	sex	age (y)	Autoimmune comorbidities	Neurological comorbidities	Vaccination status	COVID-19 severity	COVID-19–MG interval (d)	MG symptom-to-diagnosis interval (d)	MG at onset (ocular/generalized)	MG in evolution (ocular/generalized)	Antibody status	RNS	SFEMG	Chest imaging (lung/thymus)	Thymic pathology	Myasthenic crisis	Need for ICU admission	Respiratory failure	Mechanical ventilation	AChE inhibitors (pyridostigmine/neostigmine/rivastigmine)	Clinical outcome
Tugasworo et al [137]	M	60	NR	NR	NR	NR	NR	30	G (oculo-bulbar)	G	NR	NR	NR	anterior mediastinum mass suggestive of thymoma, pneumonia positive	yes, imaging	N	N	N	N	N	NR
Valjarevic et al [138]	M	57	none	none	N	NR	300	NR	G	G	AChR-Abs	decremental response	positive	NR	benign thymoma 6 years before presentation	Y	Y	Y (MG-related)	Y	neostigmine	improved
Wong et al [139]	M	78	NR	NR	NR	NR	NR	NR	G (oculo-bulbar)	G	AChR-Abs	NR	NR	NR	NR	NR	Y	Y (overlap)	Y	pyridostigmine	improved

Abbreviations: AChE- acetylcholinesterase, AChR-Abs- acetylcholine receptor antibodies, G- generalized, LRP4-Abs- low-density lipoprotein receptor-related protein 4 antibodies, MuSK-Abs - muscle-specific kinase antibodies N-no, NR- not reported, ICU- intensive care unit, O-ocular,overlap- MG and COVID-19 related, RNS- repetitive nerve stimulation, SFEMG- single-fibre electromyography, Y- yes
